# Medical Education; from N. Y. Medical Record

**Published:** 1889-07

**Authors:** 


### CULLINGS FROM CONTEMPORARIES.

### MEDICAL EDUCATION, ETC.

There can be no doubt that the feeling among the profession of this country in favor of enforcing by law, if necessary, a higher standard of medical education, is rapidly growing. It is seen in the constant reference to the subject at the meetings of medical societies, and in our current journals. The fight is between some one hundred and twenty medical colleges, and the thousand "professors" attached thereto, on the one hand, and the medical profession at large on the other. The professors have had the advantage heretofore, because they have made it their special business to kill all obnoxious legislation, while the general practitioner, having no particular and immediate interests to defend, has let matters go on as they were.

Those who, like ourselves, have been contending for higher medical standards and removal of the licensing power from the men who confer medical degrees at $30 per head, are annually helped by the statistical and other work of Dr. John H. Rauch, of Illinois. It is well to confront our readers and the public often with the figures regarding our medical men and medical institutions.

There are now in the United States about one hundred and fifteen thousand doctors, or 1 to every 533.33 inhabitants. In 1880 the ratio was 1 to 601.12. The oft-quoted ratios of other countries are as follows:

| | |
|---|---|
| Russia (in Europe) | 16 : 100,000 |
| France | 29 : 100,000 |
| Germany | 32 : 100,000 |
| Austria | 34 : 100,000 |
| United Kingdom | 61 : 100,000 |
| United States | 187 : 100,000 |

We have 118 medical colleges in this country, and the ratio of medical schools to population in this and other countries is as follows:

| | |
|---|---|
| Austria | 1 : 6,032,421 |
| Belgium | 1 : 1,384,163 |
| Denmark | 1 : 1,969,039 |
| France | 1 : 4,513,223 |
| Germany | 1 : 2,350,000 |
| Italy | 1 : 1,353,671 |
| Japan | 1 : 1,716,920 |
| Norway | 1 : 1,806,500 |
| Mexico | 1 : 1,100,890 |
| Netherlands | 1 : 1,015,142 |
| Portugal | 1 : 1,449,517 |
| Russia (in Europe) | 1 : 8,750,000 |
| Spain | 1 : 1,662,586 |
| Sweden | 1 : 1,526,300 |
| Switzerland | 1 : 948,700 |
| United Kingdom | 1 : 844,444 |
| United States | 1 : 518,846 |

With such competition among schools, and with their existence dependent upon matriculants, it is impossible that stringent requirements for graduation should be called for. Doubtless, if matters were left to take care of themselves, there would in time arise a general demand among the public for graduates of the best class of institutions. But the "supply and demand" is not entirely adequate. There will always be a majority of the people quite incapable of judging as to the qualities of a safe and wise physician. They buy the goods most obtrusively trust at them. For the protection of the public health, and for securing the greatest good to the greatest number, it is necessary to regulate the medical supply by some artificial means.

State Boards of Licensers and Examiners, with powers to prescribe the qualifications both of those who wish to become medical students, and of those who wish to become physicians, appears to furnish the best available means for promoting this end at present attainable.

The work of some such a board in Illinois, as shown in the table, offers assurance of the utility of the method:

*Comparison of the Total Number and Classes of Physicians in Practice in Illinois, July 1, 1877, and July 1, 1887:*

| | July 1, 1877. | July 1, 1887. |
|---|---|---|
| Number engaged in practice . . . . . . | 7,400 | 6,180 |
| Graduates and Licentiates . . . . . . . | 3,600 | 5,704 |
| Non-Graduates. . . . . . . . . . . . . | 3,800 | 476 |
| Percentage of Graduates and Licentiates | 48.6 | 92.3 |
| Percentege of Non-Graduates . . . . . | 51.4 | 7.7 |

A modification of the Medical Examining Board was suggested by Dr. Osler in his address before the Medico-Chirurgical Faculty of Maryland. Dr. Osler proposed "the organization of the entire profession in each State into an electorate which shall send represeentatives to a central parliament, having full control of all questions relating to medical education, examination and registration."

This means, that the doctors of each State should elect their own medical board, and, under certain legal restrictions, regulate their own affairs.

"The power of such a board," it is added, "would be accurately defined by legislation, and should relate, first, to preliminary education; secondly, to the examination and registration of candidates for the license to practice; and, thirdly, the control of all matters relating to discipline with the profession. The necessary expense would be met—first, by the fees paid by the candidates for examination; secondly, by a small annual tax levied upon all registered practitioners. Such a body could look forward hopefully to a permanent establishment in each State, with buildings suitably equipped for examination; and with every possible provision for con[illegible]ting, in an orderly and systematic manner, the business of the profession."—N. Y. Med. Record.

---

**For Sale at Half Price.**—A physician's office and gynecological chair of best make, cost $60, will sell for $30; good as new. Widow of a popular and eminent physician. Apply to editor of this journal.

